# Supplementary material for: A high-resolution mRNA expression time course of embryonic development in zebrafish
Source: eLife. 2017 Nov 16;6:e30860. doi: 10.7554/eLife.30860 (PMC5690287; doi:10.7554/eLife.30860)
Supplement: Supplementary file 6. [file elife-30860-supp6.zip › biolayout-clusters-files/Cluster116-genes.html]

Cluster116


# Cluster116: Genes

| Ensembl ID | Gene Name | Chr | Start | End | Biotype |
| --- | --- | --- | --- | --- | --- |
| ENSDARG00000089856 | CIT | 5 | 2138899 | 2372606 | protein\_coding |
| ENSDARG00000036152 | GAS2 (1 of many).1 | 7 | 32479373 | 32511297 | protein\_coding |
| ENSDARG00000016074 | cryz | 2 | 11319637 | 11335800 | protein\_coding |
| ENSDARG00000025091 | ezrb | 20 | 13672080 | 13729608 | protein\_coding |
| ENSDARG00000061752 | fam65b | 19 | 32006140 | 32120579 | protein\_coding |
| ENSDARG00000001733 | gulp1a | 9 | 42805351 | 42848392 | protein\_coding |
| ENSDARG00000098857 | lifra | 5 | 7912546 | 7978899 | protein\_coding |
| ENSDARG00000061757 | rabgap1l | 22 | 17287075 | 17429479 | protein\_coding |
| ENSDARG00000018600 | retsat | 3 | 39039529 | 39048459 | protein\_coding |
| ENSDARG00000035660 | si:ch211-193k19.1 | 19 | 44073525 | 44111878 | protein\_coding |
| ENSDARG00000091602 | si:dkey-163f14.6 | 11 | 30034957 | 30046901 | protein\_coding |
